# The Therapeutic Effect of Adding Dextromethorphan to Clonidine for Reducing Symptoms of Opioid Withdrawal: A Randomized Clinical Trial

**DOI:** 10.1155/2013/546030

**Published:** 2013-06-20

**Authors:** Ayyoub Malek, Shahrokh Amiri, Bohlool Habibi Asl

**Affiliations:** ^1^Clinical Psychiatry Research Center (CPRC), Tabriz University of Medical Sciences, Tabriz, Iran; ^2^Department of Psychiatry, Razi Psychiatric Hospital, El Goli Boulevard, P.O. Box 5456, Tabriz, Iran; ^3^Department of Pharmacology, Tabriz University of Medical Sciences, Tabriz, Iran

## Abstract

*Background*. Dextromethorphan is a noncompetitive N-methyl D-aspartate receptor antagonist that is clinically feasible for relieving the opioid withdrawal symptoms. This study compares the efficacy of a combination therapy with dextromethorphan and clonidine to treatment with clonidine alone. *Methods and Materials*. In this double-blind randomized clinical trial, patients were selected from inpatients of detox and rehabilitation ward of Razi Hospital, Tabriz, Iran. They were randomly allocated to two groups receiving either clonidine (0.4–1.2 mg/day) or clonidine and dextromethorphan (300 mg/day). Withdrawal symptoms were evaluated in the first day of admission and again 24, 48, and 72 hours later. *Results*. Thirty male patients completed the trial in each group. Withdrawal symptoms began to decrease in the second day in patients receiving dextromethorphan and clonidine while patients receiving clonidine experienced the more severe symptoms in 72 hours. Analysis of variance of the symptom severity score revealed a significant group × time interaction (*F* = 14.25; *P* < 0.001), so that patients receiving dextromethorphan plus clonidine had milder symptoms during three days in all of the measurements compared to clonidine group. *Conclusion*. Combination therapy of dextromethorphan and clonidine would result in milder opioid withdrawal symptoms compared to clonidine alone with a reduction beginning at the second day.

## 1. Introduction

Addiction is one of the most disturbing and problematic social phenomena in current years with known social, economic, and individual detriments. Unfortunately this problem has been rising in Iran during the last two decades. Documented reports have shown that there are 1200000 to 2000000 substance abusers in Iran. Mokri et al. report the number to be 4000000 abusers in 2002 [1]. These researches demonstrated that opium and heroin are the main substances being abused while achieving a complete abstinence is the main expected outcome for treatment of addiction.

Detoxification is the first step of substance abuse/dependence treatment and researches about brief and cost-effective methods receive a lot of interest in Iran [2]. Long-term opioid use would increase the cyclic Adenyl cyclase in noradrenergic system of the locus coeruleus. Hence this compensatory increase in cyclic Adenyl cyclase would result in adrenergic withdrawal symptoms during rapid opioid cessation.

Some pharmacological agents (opioid agonists, opioid agonist-antagonists, opioid antagonists, and alpha-2 agonists) are studied and used in opioid detoxification [2]. The efficacy of clonidine (as an alpha-2 agonist) in controlling the withdrawal symptoms is established in randomized placebo-controlled clinical trials [3]. A review study by Raith and Hochhaus in 2004 in Germany concludes that three main mechanisms are responsible for opioid tolerance and the withdrawal syndrome dependence which are upregulation of Adenyl cyclase and nitric oxide synthetase and activation of NMDA receptors. Consequently the use of alpha-2 agonists (e.g., clonidine) and NMDA antagonists (e.g., dextromethorphan, ketamine) may minimize the tolerance phenomenon and decrease the withdrawal symptoms [4].

The efficacy of dextromethorphan is also approved by earlier studies. A randomized clinical trial by Koyuncuoglu and Saydam in 1990 compared the efficacy of dextromethorphan and diazepam to a combination of chlorpromazine and diazepam on treating withdrawal symptoms of heroin. They showed that those patients receiving dextromethorphan and diazepam would experience milder withdrawal symptoms [5]. 

Considering the detoxification phase as the first step of the treatment of opium-addicted patients and the different effect site of NMDA antagonists (e.g., dextromethorphan) and alpha-2 adrenergic agonists (e.g., clonidine), we decided to evaluate the synergistic effects of these medications in control and relief from opiate withdrawal symptoms. Achieving success in the first step of treatment might promise further accomplishments.

## 2. Materials and Methods

This study was performed as a double-blind randomized clinical trial in Razi Hospital, Tabriz, Iran. Sixty patients, based on the results of the similar studies, were selected from among inpatients of detox and rehabilitation ward. The inclusion criteria were male sex, age range of 20 to 40 years, positive morphine test, the diagnosis of opiate dependence according to the DSM-IV-TR criteria, and mild cases of withdrawal syndrome in severity based on the Clinical Opiate Withdrawal Scale (COWS). The inclusion criteria were selected according to Gerra et al. who previously reported the interrelation of sex and the response to detoxification [6] as well as factors introduced by previous similar studies and studies about the effective factors in successful detoxification [7–9]. Also, due to medical ethics observations only mild cases of withdrawal syndrome were selected. To control the withdrawal symptoms in severe cases it was possible that we change the treatment protocol and use different dosing or add other drugs. Exclusion criteria included (1) psychotic patients (2) severe somatic diseases including hepatic or renal insufficiencies (creatinine > 1.2 mg/dL, ALT > 40 IU).

After giving a written informed consent, the selected patients, based on the inclusion and exclusion criteria, were randomly allocated to two groups receiving either clonidine alone or a combination of clonidine and dextromethorphan. For blinding the study, the examining physician who fulfilled the Clinical Opiate Withdrawal Scale (COWS) was differed from the physician who prescribed the drugs and also the prescribed drugs in one group were not clear for the patients in the other group.

The first group received clonidine 0.4–1.2 mg per day in three divided doses according to the patient's tolerance, Clonazepam 1 mg every eight hours, and acetaminophen 500 mg every six hours. Acetaminophen was used as analgesic and Clonazepam was used as a sedative and anxiolytic agent according to the standard detoxification protocols.

The second group received clonidine 0.4–1.2 mg per day in three divided doses according to the patient's tolerance, Clonazepam 1 mg every eight hours, acetaminophen 500 mg every six hours, and dextromethorphan 75 mg every six hours.

Tolerance of patients for effects of clonidine was evaluated by their blood pressure while the systolic blood pressure less than 90 mm Hg was set as the threshold for dose modification. The dextromethorphan formulation was prepared by pharmacists in Tabriz University of Medical Sciences. The other medications were selected from available preparations of the same company to obtain the same effects.

The treatment duration was four days. All of the patients were evaluated for severity of withdrawal symptoms at the admission time and 24, 48, and 72 hours later by a physician blinded to the grouping of patients. 

The Clinical Opiate Withdrawal Scale (COWS) was used by the American Association of Addiction Medicine as a good indicator of clinical severity of withdrawal symptoms [10]. COWS estimates the severity of opioid withdrawal symptoms in 11 categories: resting pulse rate, sweating, restlessness, pupil size, bone or joint aches, runny nose or tearing, gastrointestinal upset, tremor, yawning, anxiety or irritability, and gooseflesh skin. The total score is set as the indicator of severity of opioid withdrawal symptoms. The symptoms may be subdivided into four categories according to the total score as mild (5–12 scores), moderate (13–24 scores), relatively severe (25–36 scores), or very severe (more than 36 scores).

The face validity of Persian version of COWS was evaluated by some psychiatrists. As it was completed by two physicians during the study, the interobserver reliability was assessed in 18 patients and the Pearson's correlation test showed positive significant correlation between evaluators (*P* < 0.0001) with a Cronbach's alpha being 0.907 showing a high reliability. 

The study proposal was approved by Medical Ethics Committee of Tabriz University of Medical Sciences in accordance with the principles of the Declaration of Helsinki. This trial is registered with the Iranian Clinical Trials Registry (IRCT 201207196972N2).

The data was analyzed by SPSS ver.17. The continuous data are shown as mean ± SD. The principal statistical analysis evaluated the severity of the withdrawal symptoms using a 2 × 2 repeated analysis of variance (ANOVA) design with a group factor (clonidine versus clonidine and dextromethorphan), and a repeated-measures factor (pretreatment; 24, 48, and 72 hours after admission). *P* value less than 0.05 was considered significant.

## 3. Results

Thirty patients completed the trial in each group. No significant differences were observed between the two groups regarding basic characteristics including withdrawal symptoms at admission time. All were male and with Azeri ethnic background. These data are shown in Table 1. There were no clinically significant adverse events in regard to the used drugs' side effects during the trial. 

**Figure 1:**
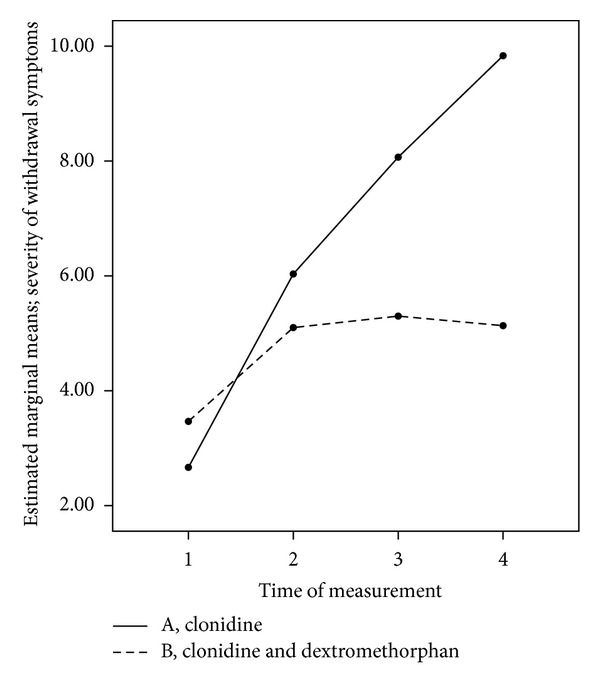
Severity of withdrawal symptoms in patients randomized to receive clonidine or clonidine and dextromethorphan measured at admission, 24, 48, and 72 hours later.

Analysis of variance of the symptom severity score (by COWS) revealed a significant group × time interaction (*F* = 14.25; *P* < 0.001), so that patients receiving dextromethorphan plus clonidine had milder symptoms during three days. As illustrated in Figure 1, symptom severity in the two groups had a different pattern and patients receiving dextromethorphan experienced milder symptoms in all of the measurements compared to clonidine group.

*At Admission (Baseline).* The withdrawal symptoms were mild at baseline (total score from five to 12 points). The withdrawal symptoms severity (mean total score) was alike between clonidine group (2.7 ± 1.9) and dextromethorphan and clonidine group (3.4 ± 2.6) with no significant difference (*P* = 0.17).

*After 24 Hours from Admission.* In both groups the withdrawal symptoms were increased compared to the baseline. This increase was more in clonidine group but without significant difference (*P* = 0.18) compared with dextromethorphan and clonidine group (6 ± 2.8 versus 5.1 ± 2.9).

*After 48 Hours from Admission.* In both groups the withdrawal symptoms were increased compared to the baseline. This increase was more in clonidine group with a significant difference (*P* = 0.0001) compared with dextromethorphan and clonidine group (8 ± 3 versus 5.3 ± 2.6).

*After 72 Hours from Admission.* The severity of withdrawal symptoms was significantly (*P* = 0.0001) more in clonidine group compared with dextromethorphan and clonidine group (9.8 ± 5.1 versus 5.1 ± 2.8).

The used drugs in this study were those used as routine agents in different diseases and had no side effects in used doses.

## 4. Discussion

This study shows that dextromethorphan added to clonidine regimen has a better efficacy compared to clonidine alone in controlling the mild withdrawal symptoms in male opioid addicts.

In the patients receiving clonidine alone the withdrawal symptoms increased across the study and reached their most severity after 72 hours from admission, while patients who received dextromethorphan and clonidine experienced milder symptoms. Their symptoms decreased after a peak in 48 hours from admission.

The alpha-2 agonists such as clonidine may decrease the withdrawal symptoms by controlling the excess activity of the adrenergic system [11]. Clonidine is being used in the treatment of withdrawal syndrome since 1980 [12–14] and a majority of detoxification regimens are using it [15], but its effectiveness is not optimal when used alone.

N-methyl-D-Aspartate (NMDA) receptors of the glutaminergic neurotransmitter system are an activator mediator of the central nervous system (CNS) and exist in different sites of CNS. Hyperstimulation of these receptors in events such as strokes, head trauma, and convulsive attacks would result in neuronal death while their decreased activity would result in schizophrenic status [11].

Kukanich and Pabich (2004) report that dextromethorphan is a noncompetitive antagonist of NMDA receptors that is used as an antitussive, adjuvant analgesic, alcohol, and opioid withdrawal symptom reducer [16]. It is also used in clinical trials combined with morphine resulting an increased analgesic effect [11]. The fact that NMDA antagonists may relieve some withdrawal symptoms is a sign of association between glutaminergic and opioid neurotransmitter systems. NMDA-antagonists would reduce the effects of opioids withdrawal on basal forebrain and amygdale, but they do not affect the locus cerelous. Moreover it seems that NMDA-antagonists would result in a delay in developing tolerance to opioid.

Another support comes from the study by Zhu et al. (2003) showing that the increased expression of C-fos gene is related to withdrawal intensity in mice neonates and dextromethorphan may reduce the related mRNA amount and is also effective in relief of withdrawal symptoms [17].

 The role of NMDA-antagonists in opioid withdrawal relief is also described for another medication from this group, memantine, which decreased the symptoms of withdrawal in heroin-addict patients [18].

A single-blind study by Bisaga et al. (1997) evaluated the role of dextromethorphan in relief of withdrawal symptoms. They used dextromethorphan (75 mg five times a day) and the result confirmed its efficacy in decreasing the withdrawal symptoms and craving. Authors suggested that it may shorten the detoxification period compared to methadone [19]. These results are confirmed by the present double-blind study as well. In a similar study (2007), it was demonstrated that memantine solely is effective in the reduction of subjective symptoms of withdrawal syndrome and the authors recommended that memantine may be used as an adjuvant therapy in the reduction of withdrawal symptoms [20].

Simultaneously there have been efforts to increase the bioavailability of dextromethorphan as well. A group of opioid dependence patients were treated with a combination of dextromethorphan and quinidine, aiming to decrease the metabolism of dextromethorphan and dextrophan level (Quinidine inhibits the metabolism of dextromethorphan, leading to reduce of dextromethorphan metabolite, dextrorphan, levels) [21]. Authors found that this combination was ineffective in reducing the withdrawal symptoms compared to placebo and most of the patients left the study in six days due to the severity of symptoms. 

In the study by Bisaga et al. dextromethorphan resulted in the reduction of withdrawal symptoms in the fourth day, which is longer than our results (reduction began in second day). This may be explained by metabolic differences of the study sample. 

This study had some limitations. The study sample consisted of patients who had decided to undertake detoxification treatment. This may decrease generalization of the results as they were highly motivated and this could influence their subjective complaints in part. However the objective symptoms could still measure their symptoms to a good extent. The double blind setting increased the accuracy as well. This study could not include a placebo group like most of the studies in this field.

## 5. Conclusion

Dextromethorphan, as a NMDA antagonist, is effective in the relief of withdrawal symptoms. Hence it is very useful in increasing the efficacy of clonidine and reducing the need for opioid agonists. Added dextromethorphan to clonidine resulted in symptom reduction as soon as the second day of admission while patients experienced very few side effects.

## Figures and Tables

**Table 1:** Severity score of opiate withdrawal symptoms as mean ± SD.

	Clonidine group	Clonidine and Dextromethorphan group	*P* value
Number of patients	30	30	—
Initial symptoms score	2.7 ± 1.9	3.4 ± 2.6	0.17
Age	29.3 ± 3.5	28.9 ± 4.3	0.56
Systolic blood pressure	125.2 ± 13.6	125.4 ± 12.5	0.67
Diastolic blood pressure	75.4 ± 10.1	74.9 ± 10.1	0.98
